# Selenocysteine β-Lyase: Biochemistry, Regulation and Physiological Role of the Selenocysteine Decomposition Enzyme

**DOI:** 10.3390/antiox8090357

**Published:** 2019-09-01

**Authors:** Lucia A. Seale

**Affiliations:** Department of Cell and Molecular Biology, John A. Burns School of Medicine, University of Hawaii, Honolulu, HI 96813, USA; lseale@hawaii.edu

**Keywords:** selenocysteine lyase, selenium, selenocysteine

## Abstract

The enzyme selenocysteine β-lyase (SCLY) was first isolated in 1982 from pig livers, followed by its identification in bacteria. SCLY works as a homodimer, utilizing pyridoxal 5’-phosphate as a cofactor, and catalyzing the specific decomposition of the amino acid selenocysteine into alanine and selenide. The enzyme is thought to deliver its selenide as a substrate for selenophosphate synthetases, which will ultimately be reutilized in selenoprotein synthesis. SCLY subcellular localization is unresolved, as it has been observed both in the cytosol and in the nucleus depending on the technical approach used. The highest SCLY expression and activity in mammals is found in the liver and kidneys. Disruption of the *Scly* gene in mice led to obesity, hyperinsulinemia, glucose intolerance, and hepatic steatosis, with SCLY being suggested as a participant in the regulation of energy metabolism in a sex-dependent manner. With the physiological role of SCLY still not fully understood, this review attempts to discuss the available literature regarding SCLY in animals and provides avenues for possible future investigation.

## 1. Introduction

### 1.1. Selenocysteine β-lyase Identification

“We have found a novel enzyme that exclusively decomposes L-selenocysteine into L-alanine and H_2_Se in various mammalian tissues and have named it selenocysteine lyase.”

The above quote was the opening sentence of the abstract of a journal article in which the enzyme selenocysteine β-lyase (SCLY) was first identified and biochemically described [1]. The same research group from Kyoto University, Japan, had previously demonstrated that the amino acid selenocysteine could be synthesized in the rat liver from selenohomocysteine, sequentially utilizing the enzymes cystathionine β-synthase (CBS) and cystathionine γ-lyase (CGL), which usually act on the transsulfuration pathway for methionine metabolism [2]. The discovery of an enzyme, SCLY, that could specifically decompose selenocysteine into L-alanine and selenide utilizing pyridoxal 5’-phosphate (PLP) as a required cofactor was, at the time, a notable puzzle piece in selenium metabolism and can be considered a landmark in the field.

This review attempts to describe and discuss what is known about SCLY, focusing further on the eukaryotic enzyme. 

### 1.2. Brief Overview of Selenocysteine and Selenoproteins

**Figure 1:**
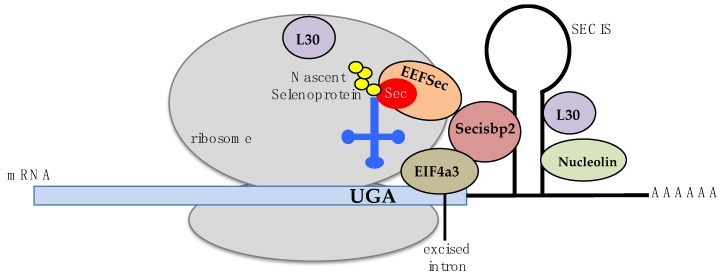
Schematic model of the UGA codon recognition and selenocysteine insertion into selenoproteins. Known participants on this molecular mechanism include proteins Secisbp2 (light red), EEFSec (orange), L30 (purple), nucleolin (green), and EIF4A3 (brown). The Sec-tRNA^Sec^ is represented in dark blue, and a nascent selenoprotein in yellow circles.

Selenocysteine, the substrate of SCLY, is an amino acid that is co-translationally incorporated into a small group of proteins called selenoproteins. Particularly, selenocysteine is encoded by the UGA codon. This codon also functions as a stop codon for protein translation; hence, specific molecular mechanisms to distinguish between translation termination and selenocysteine incorporation exist to allow for proper codon recognition. In eukaryotes, a hairpin stem-loop structure called the selenocysteine insertion sequence (SECIS) primarily present in the 3’-untranslated region of selenoprotein mRNAs is required for recoding of the triplet UGA as selenocysteine. The SECIS requires binding of the SECIS binding protein (Secisbp2) to its stem loop for the recruitment of a eukaryotic selenocysteine-specific elongation factor, EEFSec, and the ribosomal protein L30, a component of the 60S large ribosomal subunit [3,4,5,6,7,8,9]. Secisbp2 has also been revealed to function as a stabilizer of selenoprotein mRNAs [10]. Eukaryotic initiation factor 4A-III (EIF4A3) and nucleolin are proteins also participating in SECIS recognition of the UGA codon via binding to Secisbp2 or to the SECIS element. However, details of their involvement in this process remain unclear [11,12,13]. Selenocysteine insertion into a peptide chain also requires a distinct selenocysteine tRNA, the Sec-tRNA^[Ser]Sec^, containing the anticodon ACU. Biochemical modifications of the Sec-tRNA^[Ser]Sec^ encompass a pseudouridine at position 55, an isopentenyladenosine at position 37, a 1-methyladenosine at position 58, and a 5-methoxycarbonylmethyluridine at position 34 that is methylated in selenium adequacy, with this methylation step regulated by the presence of a transcription factor, STAF, binding region [14,15,16]. These modifications confer specificity of the Sec-tRNA^[Ser]Sec^ to the UGA recognition, promoting selenocysteine incorporation into selenoproteins. A mechanistic model for SECIS recognition of the UGA codon has been suggested [17,18] and is briefly summarized in Figure 1.

There are 24 and 25 genes for selenocysteine-incorporated selenoproteins in mice and humans, respectively [19]. Selenoproteins are distributed in all kingdoms of life and are essential in mammals. Often, the selenocysteine residue is in the active site of a selenoprotein, except for selenoprotein P (SelenoP), which contains several selenocysteine residues and functions as a selenium transporter throughout the bloodstream [20]. The selenium atom of selenocysteine is easily oxidized into selenolate forming selenocystine. Moreover, selenium has a large atomic radius, being able to act as both electrophile and nucleophile, with reaction rates 2–4 orders of magnitude higher than sulfur. This occurs as selenocysteine is found deprotonated at physiological pH due to its lower pK*_a_* of 5.43 when compared to a pK*_a_* of 8.22 to the sulfur-analog amino acid, cysteine [21]. The increased reactivity of the selenolate exchange with diselenides and disulfides warrants selenoproteins to be mostly involved in redox reactions, curbing oxidative stress [7]. Selenocysteine can then be used by proteins as a reactive handle, enabling transamidation of peptide segments, metal-catalyzed reactions, and generation of dehydroalanine when in the presence of peroxides [22]. Formation of dehydroalanine is possibly an additional mechanism for modulating a cellular redox state, as it can, in turn, inactivate a selenoprotein [23]. 

### 1.3. Biosynthesis of Selenocysteine 

Selenocysteine to be incorporated into proteins is biosynthesized on its own tRNA (Sec-tRNA^[Ser]Sec^). Primarily, the Sec-tRNA^[Ser]Sec^ is aminoacylated with serine by seryl-tRNA synthetase and then phosphorylated by the enzyme phosphoseryl-tRNA^[Ser]Sec^ kinase (PTSK), forming the intermediate *O*-phosphoseryl-tRNA^[Ser]Sec^ [24,25]. This step is followed by conversion of the serine moiety to selenocysteine by the enzyme selenocysteine synthase (SecS), producing the Sec-tRNA^[Ser]Sec^. SecS provides a selenophosphate for the exchange [26]. Prior production of this selenophosphate stems from selenide, which is delivered to the phosphate moiety of selenophosphate synthetase enzymes (SEPHS1 and SEPHS2, the latter, itself, a selenoprotein). SEPHS2 has been demonstrated to be required for selenoprotein biosynthesis [27], while SEPHS1 is involved in the maintenance of cellular redox homeostasis [28,29]. Interestingly, selenium deficiency may also allow for cysteine loading of the tRNA^[Ser]Sec^ with consequent formation of thiophosphate and insertion of cysteine instead of selenocysteine into UGA sites [30,31]. 

**Figure 2:**
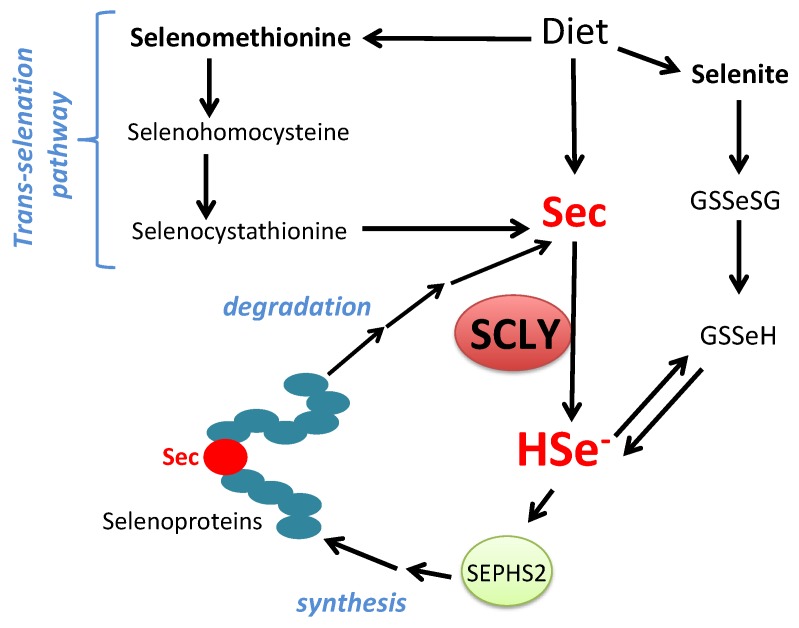
Schematic view of SCLY participation in selenium metabolism. Sec, selenocysteine; HSe^-^, selenide; GSSeSG, selenodiglutathione; GSSeH, selenoglutathione; SEPHS2, selenophosphate synthethase 2.

The amino acid selenocysteine can be acquired through diet, released after selenoprotein degradation or produced inside cells as a byproduct of selenomethionine metabolism via the trans-selenation pathway. Dietary selenocysteine is found in animal products and possibly corresponds to a small percentage of available selenocompounds in plant-based foods [32]. Ingested dietary selenocompounds are absorbed by the intestines. Interestingly, selenocysteine acquired either from dietary sources, released from selenoprotein degradation or produced via the trans-selenation pathway still needs to be decomposed into selenide by SCLY to be used in selenoprotein translation [33]. This occurs as the enzyme SEPHS2 specifically utilizes selenide to produce monoselenophosphate for selenocysteine biosynthesis. Such a particularity generates a critical recycling step, by which selenocysteine is broken down to be precisely resynthesized for exclusive use in selenoprotein translation. Figure 2 summarizes the role of SCLY in these pathways and the recycling mechanism.

## 2. Selenocysteine β-Lyase

### 2.1. Biochemical Characteristics and Mechanism of Action

SCLY is a member of class V of pyridoxal 5’-phosphate PLP-dependent aminotransferases [34,35] that share an evolutionarily conserved domain in which a lysine residue binds to the cofactor PLP for homodimerization. One unit of SCLY has a molecular weight of approximately 48,000, varying according to the species. Nevertheless, SCLY functions as a homodimer, and it was first detected in pig liver with an approximate molecular weight of 93,000 using a Sephadex G-200 gel filtration methodology [1,36]. The homodimer assembly forms an active site pocket, where L-selenocysteine binds, as well as a secondary pocket for the binding of cofactor PLP. 

The close structural similarity between L-selenocysteine and L-cysteine suggested that SCLY could use both amino acids as substrates, as do cysteine desulfurases. Nevertheless, in terms of its substrate recognition, having ruled out SCLY action on other amino acid substrates as well as glutathione, it was established that the pig enzyme specifically acted on a selenocompound, L-selenocysteine, with a K*_m_* of 0.83 mM, while the K*_i_* for competitor L-cysteine was 1.0 mM [1]. For human SCLY, the K*_m_* for L-selenocysteine was determined to be 0.5 mM with a K*_i_* for L-cysteine of 5.85 mM [37], while for mouse Scly, the K*_m_* for L-selenocysteine is 9.9 mM [34,38]. Because the K*_m_* value for L-selenocysteine as a substrate was much higher than expected concentrations of this amino acid in cells, it is possible that SCLY works slowly in vivo, even having an alternative physiological role apart from selenocysteine decomposition. Yet, rat SCLY can also decompose selenium-methylated compounds, such as selenium-methylselenocysteine, and releasing, after a demethylase step, selenide and methanol as final products [39].

**Figure 3:**
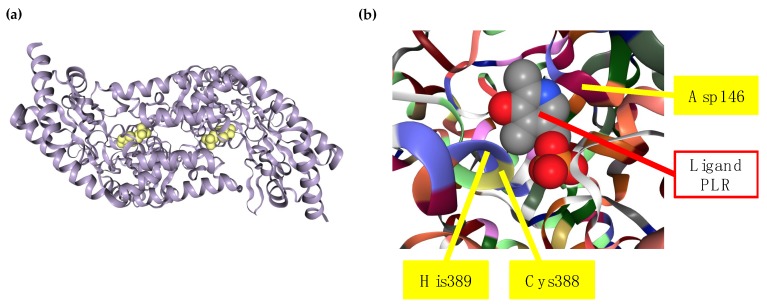
**Human SCLY structure.** The gray cartoon structure of human SCLY in (**a**) represents the homodimer enzyme with ligand PLR bound (space filled in yellow) to experimentally demonstrate the PLP pocket in SCLY. (**b**) is a close-up view of the active site of human SCLY, with each amino acid residue in one color, and pointing with the yellow bars to critical residues Cys388 (yellow), His389 (purple), and Asp146 (chianti). Space filled ligand, PLR, is shown in gray, red, and blue. The human SCLY structure was obtained from the Protein Data Bank (PDB) Japan entry number 3GZC, deposited by Collins et al. [41], and using the Research Collaboratory for Structural Bioinformatics (RSCB) PDB online tool (rscb.org) [43].

The crystal structure of rat and human SCLY have been determined, as well as the molecular mechanism behind their specificity to L-selenocysteine and its capacity to discriminate from sulfur amino acids. Each subunit of SCLY consists of a small and a large domain, with two active site cavities at the interface of the subunits, and an extended lobe that is disordered without ligand and becomes ordered in the presence of the ligand. Besides L-selenocysteine, both L-cysteine and D-selenocysteine are able to enter the active site of SCLY but cannot serve as substrates to the enzyme. This incapacity occurs because, upon binding of L-selenocysteine in the active site, this substrate is deprotonated and forms a sulfoselenide intermediate, which does not form with the other substrates. A critical cysteine residue at position 388 (Cys388; equivalent to Cys375 in rat Scly) in the active site of human SCLY binds to L-selenocysteine, forming a selenoato-thiol interaction and a Schiff base with PLP that results in protonated aldimine production. An aspartate residue at position 146 (Asp146) of human SCLY is found in close proximity to the crucial Cys388 residue and is also considered essential for the substrate specificity towards L-selenocysteine. Moreover, a histidine residue at position 389 (His389) in the human SCLY also influences the activity of the enzyme due to the close proximity to the Cys388 and Asp146 residues in its tridimensional structure [40,41,42]. The human SCLY structure bound to a 4’-deoxypyridoxine phosphate (PLR) ligand (mimicking PLP) as well as the map of critical amino acid residues for L-selenocysteine specificity is represented in Figure 3.

Neither SEPHS enzymes nor SecS, the sequential enzymes for selenocysteine biosynthesis for selenoproteins, are able to differentiate between selenium and sulfur compounds. Therefore, SCLY has been suggested to be an important sorter of the process of selenocysteine biosynthesis for selenoprotein production [40]. Nevertheless, biochemical confirmation that SCLY directly delivers selenide to SEPHS2 in eukaryotes remains unreported, as does the identification of a potential selenium-transferring protein specific for this task. Based on the available data, however, SCLY is currently the best candidate for that function.

### 2.2. Phylogenetic Distribution

The SCLY gene is highly conserved in animals. Alignments of vertebrate sequences with the mouse *Scly* cDNA sequence revealed orthologous genes in humans, chickens, and zebrafish. The National Center for Biotechnology Information (NCBI) gene database currently lists 286 organisms containing orthologs of the human SCLY gene [44]. Moreover, *Scly* homologous genes were evident in invertebrates, such as cnidarians, nematodes, and sea squirts [45]. A phylogenetic tree generated for cysteine desulfurase Nfs-like genes using 150 genomes revealed a eukaryotic clustering of sequences for *Scly*, including from single-cell organisms such as *Trypanosoma brucei* and *Toxoplasma* sp [46].

Searching for a homologous SCLY gene in bacteria, archaea, fungi, and plants ended unsuccessfully. However, bacteria and algae have selenoproteins and selenium-dependent enzymes, and hence, these organisms are able to metabolize selenium and incorporate selenocysteine [47,48,49,50]. Yet, the absence of a SCLY gene is somehow puzzling. Such a paradox may stem from the microbial dissimilatory reduction of the oxyanions, selenate and selenite, which are highly toxic, into elemental selenium, which is non-toxic and less bioavailable. Elemental selenium serves as a microbial immobilized storage of selenium as nanoparticles, a product being utilized in bioremediation and biotechnology [51,52,53,54]. Elemental selenium can also be further reduced to selenide for direct utilization in the production of selenophosphate for selenocysteine synthesis in bacteria [55]. With a source of selenide to withstand selenoprotein translation, microbial organisms can circumvent the absence of a bona fide SCLY. An additional unlikely possibility is, however, that the enzyme performing selenocysteine decomposition in these organisms has a highly distinct primary sequence yet possibly maintains the tertiary structure.

On the other hand, selenocysteine β-lyase activity was detected in several bacterial and fungi species, such as *Pseudomonas alkanolytica*, *Alcaligenes viscolactis,* and *Escherichia freundii* [56]. Interestingly, in bacteria, three enzymes have been identified with selenocysteine **β**-lyase activity, the *Azobacter vinelandii* Nfs protein and the *Escherichia coli* cysteine desulfurases (CsdA and CsdB) [38,57,58,59]. Moreover, a selenocysteine lyase/cysteine desulfurase regulated by selenite levels was identified in the probiotic organism, *Lactobacillus reuteri* [60]. These enzymes are PLP-dependent and can indiscriminately decompose L-cysteine and L-selenocysteine into alanine and elemental sulfur and selenium, respectively. In the absence of the cofactor PLP, these enzymes are inactivated by abortive transamination, yielding pyruvate. Nevertheless, the specificity by which Csd and Nfs enzymes recognize either cysteine or selenocysteine as a substrate differs [61] and might play a regulatory role in selenium metabolism in these organisms. The selenocysteine lyase function is likely performed by closely related enzymes such as CsdB and Nfs in a non-specific manner.

Strikingly, the primary structure of SCLY shares ~30% sequence identity with Nfs-type cysteine desulfurases. These enzymes decompose the amino acid L-cysteine into alanine and sulfide, providing sulfur to iron-sulfur clusters, tRNA thionucleotides, and thiamine biosynthesis [62]. It is notable that the parasitic protist, *Trypanosoma brucei*, also has an Nfs-protein that presents selenocysteine β-lyase activity; however, SCLY in *T. brucei* does not have cysteine desulfurase activity [46].

### 2.3. Subcellular Localization

The subcellular localization of SCLY is a controversial topic. Based on the function the enzyme possesses and the absence of a recognizable signaling peptide, it is logical to infer its localization would be restricted to the cytosolic compartment. In fact, SCLY was mostly detected in the cytosol after subcellular fractionation of the mouse liver [34]. Additionally, the same study detected SCLY in the mitochondria, in microsomes, and in the nuclear compartment, although in less quantity. A study using *Trypanosoma brucei* also uncovered that, after subcellular fractionation of tetracycline-induced cells, most hemagglutinin-tagged SCLY expression was found in the cytosolic fraction [46]. 

Intriguingly, when immunofluorescence microscopy was used to detect SCLY in kidneys, testis, and livers of mice, the enzyme was predominantly detected in the nucleus [33]. The same pattern of nuclear localization upon immunofluorescence was observed in *Trypanosoma brucei* [46]. It is highly intriguing that SCLY is predominantly found in the nucleus in immunofluorescence preparations, and currently, the role of this enzyme, when localized in the nucleus, is unknown. Sorting which specific chemical or temporal conditions allowing for the divergent subcellular distribution of SCLY, as well as the molecular mechanism that transports the protein to the nucleus, will possibly shed light on additional roles and regulatory pathways in which SCLY may be involved.

### 2.4. Tissue Distribution of SCLY

The enzyme SCLY was first purified from pig liver, and its presence has been detected in other mammalian tissues, including kidneys, pancreas, adrenal, spleen, testis, brain, heart, muscles, and lung, while absent in epididymal white adipose tissue and blood [1,63,64,65]. Expression of *Scly* mRNA in mice has been observed in the liver, kidneys, testis, heart, stomach, brain, and lung, with the highest expression in the liver [34,63]. In silico analysis of the human transcriptome has uncovered SCLY mRNA expression in the same tissues, with the highest expression also in the liver and kidneys [66,67].

It is worth note that, comparatively, euryhaline tilapia (*Oreochromis mossambicus*) expresses *Scly* mRNA in the gills, kidney, liver, pituitary, and brain, with the highest expression in the gills regardless of environmental salinity [68]. As fish gills are under significant oxidative stress coming from the surrounding environment, such high expression suggests that SCLY may be valuable for detoxification mechanisms in this tissue, possibly by providing selenide for the synthesis of selenoprotein(s) directly involved in curbing oxidative stress such as glutathione peroxidases (GPx). 

### 2.5. Regulation of Gene Expression and Protein Levels

The human SCLY gene resides on chromosome 2 at position 2q37.3, while the mouse *Scly* gene is found on chromosome 1 of the genome, flanked in both genomes by the same genes. Both human and mouse SCLY genes produce a pre-mRNA with 12 exons. The 5’-regulatory region of the rat SCLY gene has an enhancer element between –152 and –298, and this enhancer encompasses two AP-1 binding sites and one binding site for transcription factor NFκE [69]. Moreover, the *Scly* promoter has two other AP-1 binding sites between position –500 and –1000. AP-1 sites bind dimeric variations of transcription factors Jun and c-Fos, which are responsive to oxidative stress and pro-inflammatory cytokines [70]. The occurrence of these binding sites suggests that SCLY expression is regulated by these conditions. 

Interestingly, in a mouse model lacking the regenerating islet-derived 3 beta (Reg3β) protein, only the c-Fos subunit of AP-1 was reported to activate *Scly* transcription. The transcription factor c-Fos bound to two of the four AP-1 binding sites in the *Scly* promoter—the most proximal one and the other close to the –500 position. Lack of Reg3β also enhanced selenoprotein biosynthesis, particularly of GPx1, and nitration levels in the liver, with Scly being one of the nitration targets. The overall enhanced nitration observed in this mouse model probably triggered the transcriptional activation of *Scly*, allowing for the upregulation of selenoproteins to compensate for the oxidative stress [70].

Hypoxic conditions were also revealed as downregulators of the SCLY mRNA in human hepatocellular carcinoma (HepG2) cells. Interestingly, SCLY mRNA expression in these cells was upregulated by treatment with 100 nM selenite, but the downregulatory effect of hypoxia occurred regardless of selenium levels. Moreover, such a downregulatory effect was independent of hypoxia-inducible factors (HIFs) classically involved in response to hypoxic conditions, suggesting an alternative route of regulation of the selenoprotein synthesis machinery by hypoxia. Notably, gene expression of SEPHS2 was also severely downregulated by chronic hypoxia, a correlation that suggests limiting Sec-tRNA^[Ser]Sec^ loading is occurring, which could explain the downregulation of most selenoproteins observed [71]. Interestingly, infection of the same cell line with the core protein of the hepatitis C virus revealed SCLY to be the gene most differentially expressed using a differential display RT-PCR technique [72]. Since hepatocellular carcinoma after chronic infection with hepatitis C virus is a condition known to decrease cellular availability of selenium [73], it could be possible that SCLY is responding to diminished selenium levels after infection, and not to the viral infection itself.

Paradoxically, however, SCLY activity was reported as not regulated by selenium levels or the chemical form of selenium provided in the diet. Feeding rats for nine weeks with 2 ppm of either selenite, selenocysteine, or selenomethionine did not result in significant changes in activity levels of SCLY in liver, kidneys, testis, or muscle [65]. On the other hand, mice fed for eight weeks with diets containing either 0.08, 0.25, or 1 ppm of selenite showed an inverse regulation of *Scly* mRNA expression in the liver and brain [63], suggesting that selenium levels affect gene expression, with other molecular mechanisms independent of selenium possibly curbing the enzyme activity. 

In terms of the regulation of *Scly* gene expression by endocrine factors, it was reported that glucocorticoids negatively regulate *Scly* mRNA in mice. A study that performed RNA-sequencing analysis of the hypothalamic arcuate nuclei of mice chronically treated with corticosterone revealed a remarkable downregulation of the *Scly* gene in this region. Strikingly, in the first two days of glucocorticoid treatment, the mRNA for two selenoproteins, *SelenoP,* and iodothyronine deiodinase 2 (*Dio2*), were upregulated [74]. The enzyme DIO2 a key regulator of thyroid hormone activation in cells, particularly in the hypothalamic-pituitary axis [75,76]. Upregulation of the *Dio2* gene in mice was maintained upon chronic treatment with corticosterone [74], implying that, expression or activity of this selenoprotein could be dependent on the absence of SCLY.

A study revealed the SCLY transcript to be upregulated on the fourth day after treatment with a gonadotropin-releasing hormone (GnRH) agonist, buserelin. This upregulation occurred at the uterine endometrial tissue of Nelore cows with large preovulatory follicles and corpus luteum, which are an indication of greater receptivity after in vitro fertilization protocols [77]. GnRH is a hypothalamic hormone, released to activate the production and secretion of follicle-stimulating hormone (FSH) and the luteinizing hormone (LH) in the anterior pituitary. It is still unclear whether the upregulation of SCLY transcript in the uterine endometrial tissue is a direct or indirect regulatory response to either the GnRH agonist treatment or one of the downstream reproductive hormones via a negative feedback mechanism. Another study revealed that the SCLY gene was upregulated post-oestrus in pubertal gilt livers and kidneys after dietary intake of selenium-enriched yeast with vitamin B6 supplementation. This potentially occurred as a response for hormonal changes of this period and involvement of SCLY in the synergistic regulation with vitamin B6 of the GPx system via the trans-selenation pathway [78].

Interestingly, renal SCLY expression in tilapia (*Oreochromis mossambicus*) was inversely correlated with environmental salinity [68]. This correlation implies that the SCLY gene in fish may be regulated either by the salt concentration or one of the endocrine factors that coordinate the osmoregulatory response in fish, such as prolactin [79].

### 2.6. Physiological Role

SCLY has been proposed to provide selenide for the biosynthesis of selenoproteins [34], and hence, has become an important player in selenium metabolism, particularly for selenoprotein biosynthesis. To participate as a selenide provider, SCLY is postulated to transfer selenide to the selenophosphate synthetase enzymes SEPHS1 and SEPHS2 [40,62]. These enzymes are responsible for selenite assimilation and selenocysteine recycling, respectively [80]. SEPHS2 possibly uses the selenide for the production of selenophosphate, a required step in the synthesis and charging of the selenocysteine amino acid to its specific tRNA^[Ser]Sec^ as described in Section 1.3 of this review. Molecular interaction between SCLY and both SEPHS1 and SEPHS2 has been demonstrated in vitro [81], but not in vivo. 

Importantly, the involvement of SCLY in the biosynthesis of selenoproteins was confirmed using RNAi technology. HeLa cells depleted of SCLY had decreased expression of selenium-sensitive selenoprotein GPx1. This effect in GPx1 was rescued by treatment with selenite or selenomethionine, but not with selenocysteine, confirming the necessity of SCLY-dependent decomposition of selenocysteine for the synthesis of GPx1. Interestingly, when supplemented with SelenoP as a selenium source, SCLY-depleted HeLa cells showed a striking decrease in selenoprotein biosynthesis, suggesting that the selenocysteine utilized by SCLY possibly comes from the degradation of SelenoP [33]. Mice lacking the *SelenoP* gene had also decreased gene expression of *Scly*, however, without affecting either Scly protein levels or activity. Moreover, the effect on *Scly* gene expression was only observed in the liver, suggesting that selenocysteine provided by the degradation of SelenoP is not crucial for the role of Scly in the physiology of most tissues [63].

Nevertheless, selenide provided by recycling mechanisms after selenoprotein degradation for selenoprotein synthesis is also essential for neurological health, as male mice lacking both *Scly* and *SelenoP* genes had a shortened lifespan with severe seizures upon the onset of puberty. Such detrimental effect was eliminated when animals were either supplemented with selenium or castrated [82,83]. 

Mice lacking the *Scly* gene (*Scly^−/−^*) had only mild effects on their neurological health, mostly when challenged with a selenium-deficient diet [84]. This outcome suggested that their brains possibly activate additional mechanisms for obtaining selenium for selenoproteins, such as selenite reduction, not relying solely on selenocysteine decomposition. Intriguingly, however, the same mouse model was prone to the development of obesity with glucose intolerance, hyperinsulinemia, insulin resistance, and hepatic steatosis, a phenotype that was exacerbated under selenium-deficient conditions [64]. This observation suggested the participation of SCLY in energy metabolism, particularly at the liver where the enzyme is most active. Male *Scly^−/−^* mice also worsened their obesity when fed a high-fat diet, even when dietary selenium levels were adequate [85], suggesting that the role of SCLY in energy metabolism could be independent of its involvement in selenoprotein biosynthesis. On the other hand, female *Scly^−/−^* mice had an attenuated phenotype, gaining weight and accumulating lipid in their fat depots but without the effects on glucose homeostasis and insulin levels. Interestingly, castration of male *Scly^−/−^* mice restored insulin levels and energy expenditure outcomes, a result that suggests a physiological role for selenocysteine decomposition in the testis in improving metabolic outcomes [86].

Selenium metabolism is strongly affected by sex [87,88]. Hence, it was not surprising to observe a sexual dimorphism in the phenotype of *Scly^−/−^* mice [64,86]. Remarkably, these sex-dependent differences were not due to a central regulatory role of Scly, as both male and female *Scly^−/−^* mice equally diminished levels of stress-regulated selenoproteins GPx1, selenoprotein M (SelenoM), and selenoprotein S (SelenoS) in the hypothalamus [86]. The hypothalamic selenoprotein downregulation in both sexes suggests that distinct, sex-independent molecular mechanisms are in place to regulate the synthesis of selenoproteins in a compartmentalized manner at the central level. The hypothalamus is a neuroendocrine regulator of energy balance, mostly via the neuronal outputs from the arcuate, ventromedial, preoptic area, and paraventricular nuclei. These hypothalamic structures act on critical aspects of energy homeostasis, such as feeding behavior and brown fat thermogenesis [89,90,91]. Intriguingly, targeted deletion of the *Scly* gene in Agrp neurons of the arcuate nucleus of the mouse hypothalamus led to protection against diet-induced obesity with an elevation of brown fat thermogenesis marker uncoupling protein 1 in both sexes [92]. Therefore, the sexually dimorphic effects on metabolic health observed in the whole-body *Scly^−/−^* mice suggest that peripheric tissues are sorting and coordinating mechanisms to compensate for hypothalamic suppression of stress-related selenoprotein synthesis. Moreover, compensatory mechanisms are likely also regulated by sex, leading to impairment in glucose and lipid homeostasis, and inducing a metabolic syndrome-like phenotype exacerbated in the males of the *Scly*^−/−^ mouse model. Overall, the results obtained in mouse models lacking the *Scly* gene were able to shed light on the role of this enzyme as an important player in hypothalamic synthesis of selenoproteins and the regulation of hepatic insulin-dependent glucose homeostasis, broadly connecting selenocysteine decomposition to the physiological regulation of energy metabolism.

Supporting a role for SCLY in coordinating the intersection between selenium and energy metabolism, at least in the liver, is the evidence that SCLY interacts with several proteins involved in metabolism. Yeast two-hybrid screening studies using mouse Scly as protein bait revealed an array of interactors with diverse functions such as major urinary proteins (MUPs), aldehyde reductase, ATP synthase A, glutathione S-transferase, farnesyl diphosphate farnesyl transferase I, vitamin D-binding precursor, liver regeneration p-53 related protein, lysosomal pepstatin-insensitive protease, and apolipoprotein A-II, among others [81,93]. Interestingly, interactors specifically involved in protein synthesis were also revealed, such as TATA box binding protein-like 1, RAN binding protein 9, splicing factor arginine/serine-rich 5, nucleotide binding protein-like, and RNA helicase. Except for MUP-1 [93], all other potential interactors have not been validated either in vitro or in vivo. In addition, the yeast two-hybrid approach has also not identified a direct interaction of mouse Scly with any of the SEPHS enzymes, and this interaction was revealed through directed in vitro studies only [81]. The diverse list of interactors suggests that Scly may participate in additional functional pathways beyond what is known, described participation in selenoprotein synthesis via selenocysteine decomposition. Moreover, nuclear-resident interactors may provide valuable insight into the role of SCLY when localized in the nucleus.

### 2.7. SCLY Gene Polymorphisms 

There are currently 530 single nucleotide polymorphisms (SNPs) mapped in the human SCLY gene according to the SNP database from the National Institutes of Health of the United States of America. Among all these SNPs, only two of them have been connected with a disease condition. A genome-wide linkage and association (GWAS) analysis in Americans of Mexican origin as part of The Insulin Resistance Atherosclerosis Family Study uncovered two SCLY SNPs associated with cholesterol levels. SNP rs201606363 is in the intronic area of the SCLY gene, while SNP rs3832063 is found in the intergenic area between the gene for UBE2F and SCLY. Both ranked highest in the GWAS analysis and were determined to be associated with low-density lipoprotein and cholesterol levels [94]. 

## 3. Conclusions

Since the first description of SCLY in 1982, several aspects of its biochemistry and biology have been elucidated. Nevertheless, significant questions remain. For instance, the subcellular localization of SCLY is still unresolved, and sorting this issue will improve our understanding of selenium metabolism and the enzyme role on it. Another unresolved issue is the particular mechanism of selenium delivery to SEPHS enzymes, as it is not currently known whether SCLY directly provides selenide to selenophosphate synthesis, or if an intermediate protein exists that receives the SCLY-produced selenide and delivers to SEPHS. 

Yet, as it is becoming apparent with recent studies, SCLY is possibly involved in aspects of energy metabolism, particularly in the liver. The fact that the enzyme might act as a bridge between selenium metabolism and how carbohydrate and lipid are handled in cells over different conditions is an element of the role of SCLY that needs to be more extensively sought. The impact of the actions of SCLY in human health and nutrition, particularly to matters where selenium is already known to be involved, may bring an enriched perspective on overall physiology, potentially uncovering novel molecular mechanisms dependent on SCLY.

## Abbreviations

CBS: cystathionine beta synthase; CGL, cystathionine gamma lyase; Csd, *E. coli* cysteine desulfurase; DIO2, iodothyronine deiodinase 2; EFSec, selenocysteine-specific elongation factor; FSH, follicle-stimulating hormone; GnRH, gonadotropin-releasing hormone; GPX, glutathione peroxidase; GSSeSG, selenodiglutathione; GSSeH, selenoglutathione; GWAS, genome-wide linkage and association; HSe^-^, selenide; LH, luteinizing hormone; MUP, major urinary protein; Nfs, cysteine desulfurase; PLP, pyridoxal 5’-phosphate; PTSK, phosphoseryl-tRNA^[Ser]Sec^ kinase; Reg3β, regenerating islet-derived 3 beta; Secisbp2, SECIS binding protein; SCLY, selenocysteine lyase; Sec, selenocysteine; SECIS, selenocysteine insertion sequence; SecS, selenocysteine synthase; SelenoM, selenoprotein M; SelenoP, selenoprotein P; SelenoS, selenoprotein S; SEPHS, selenophosphate synthethase; SNP, single nucleotide polymorphism.
